# Protocatechualdehyde Induces S-Phase Arrest and Apoptosis by Stimulating the p27^KIP1^-Cyclin A/D1-CDK2 and Mitochondrial Apoptotic Pathways in HT-29 Cells

**DOI:** 10.3390/molecules21070934

**Published:** 2016-07-19

**Authors:** Shi Zhong, You-Gui Li, Dong-Feng Ji, Tian-Bao Lin, Zhi-Qiang Lv

**Affiliations:** Sericultural Research Institute, Zhejiang Academy of Agricultural Science, Hangzhou 310021, China; zshi2002@163.com (S.Z.); liyougui3@163.com (Y.-G.L.); tianbao_lin@126.com (T.-B.L.); lvzhiqiang_3@126.com (Z.-Q.L.)

**Keywords:** *Phellinus gilvus*, protocatechualdehyde, S-phase arrest, apoptosis, HT-29 cells

## Abstract

Protocatechualdehyde (PCA) extracted from *Phellinus gilvus* exhibits anti-cancer activity in human colorectal carcinoma cells (HT-29). However, the underlying mechanisms remain poorly understood. We performed an in vitro study involving MTT, flow cytometry, RT-PCR, and western blot analyses to investigate the effects of PCA treatment on cell proliferation, cell cycle distribution, apoptosis, and expression of several cell cycle-related genes in HT-29 cells. The treatment enhanced S-phase cell cycle and apoptosis in HT-29 cells in a dose-dependent manner. Western blot results showed that PCA treatment decreased the expression levels of cyclin A, cyclin D1, and p27^KIP1^ but increased those of cyclin-dependent kinase 2 (CDK2) in HT-29 cells. Furthermore, the expression levels of B-cell lymphoma/leukemia-2 (Bcl-2) and B-cell lymphoma/leukemia-xL (Bcl-xL) were down-regulated, whereas the levels of BH3-interacting domain death agonist (Bid), Bcl-2 homologous antagonist/killer (Bak), and cytosolic cytochrome c were significantly upregulated. Thus, the enzymes caspases-9, -3, -8, and -6 were found to be activated in HT-29 cells with PCA treatment. These results indicate that PCA-induced S-phase cell cycle arrest and apoptosis involve p27^KIP1^-mediated activation of the cyclin-A/D1-Cdk2 signaling pathway and the mitochondrial apoptotic pathway.

## 1. Introduction

**Figure 1:**
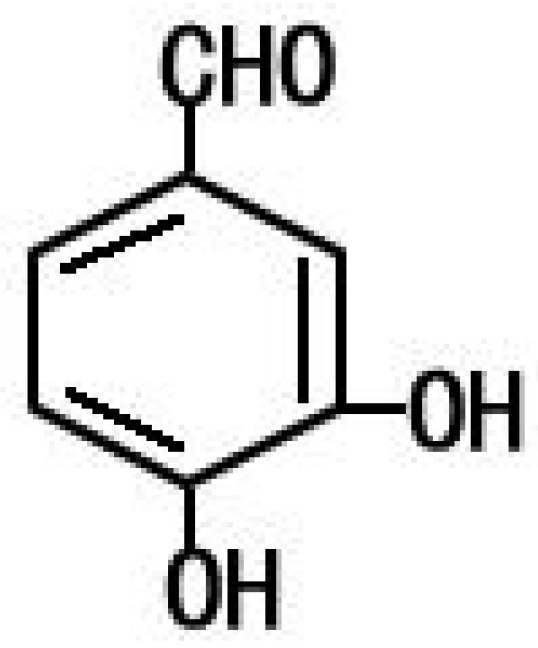
Structure of protocatechualdehyde (PCA).

Phytochemicals from natural resources exhibit strong potential for use in cancer treatment. *Phellinus spp* (Mesima) is a basidomycota fungus that is rich in polysaccharides and polyphenol compounds. It has been used in traditional medicine for over 2000 years to treat various diseases [1,2,3]. Several lines of research have indicated that *Phellinus spp* possesses immunomodulating [4], antitumor [5,6], and anti-inflammatory activities [7]. Protocatechualdehyde (PCA, Figure 1) is one of the main polyphenols extracted from *Phellinus gilvus* [8]. PCA exhibits anti-cancer activities in human breast cancer cells [9] and leukemia cells by inhibiting casein kinase II activity [10]. In addition, it downregulates cyclin D1 and HDAC2, and activates ATF3 expression in human colorectal cancer cell lines, namely, HCT116 and SW480 cells [11,12]. Our previous study reported that PCA can inhibit the proliferation of HT-29 cells [8]. However, the mechanisms by which PCA inhibits HT-29 cells have not been studied so far.

Aberration in cell cycle regulation constitutes a key mechanism underlying cancer cell growth [13,14,15]. Many natural agents can cause cell cycle arrest at different phases and induce apoptosis [16]. Achieving a balance between cell cycle arrest and cell death induction is critical for the efficacy of anticancer drugs. PCA attenuates cyclin D1 expression, resulting in inhibition of cell growth and apoptosis in HCT116 and SW480 cells [11,12]. Therefore, controlling the expression of genes that regulate PCA-mediated cell cycle could form the basis of both preventive and therapeutic measures against cancer. To study the mechanisms underlying the therapeutic properties of PCA, we examined the effects of this compound on cell cycle distribution, apoptosis, and expression of several regulatory proteins and signaling molecules in HT-29 cells. Our results provide the first evidence that PCA inhibits HT-29 cell proliferation through S-phase arrest and apoptosis by regulating the p27^KIP1^-cyclin A/D1-CDK2 and mitochondrial apoptotic pathways.

## 2. Results

### 2.1. Effect of PCA on HT-29 Cell Proliferation

**Figure 2:**
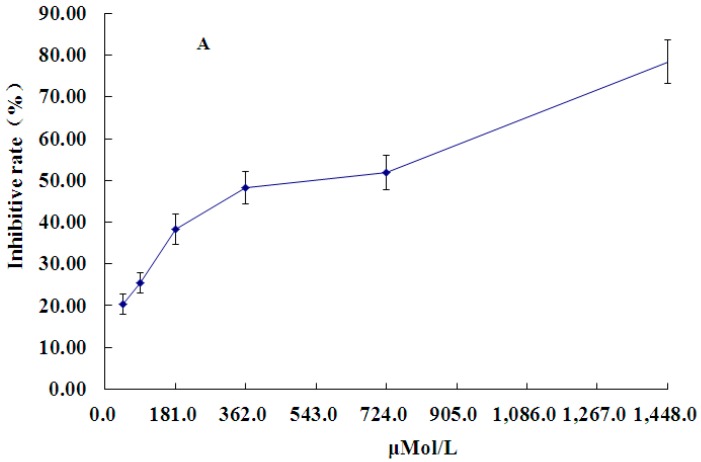

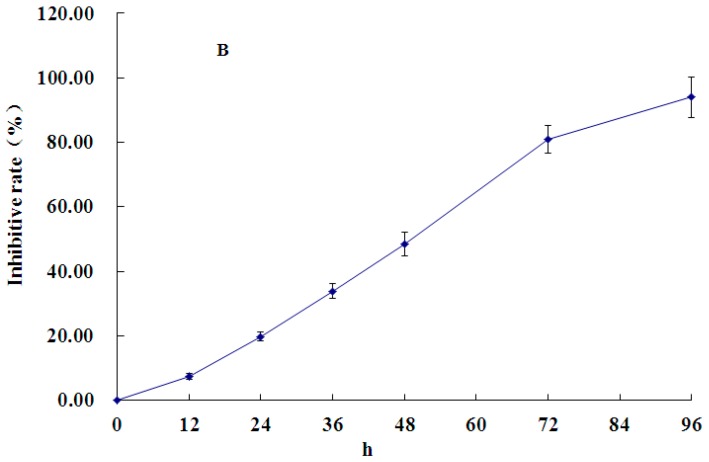
Inhibitory effect of PCA on HT-29 in vitro. (**A**) HT-29 cells were treated with various concentrations of PCA (45–1448 μM) and cultured up to 48 h; (**B**) HT-29 cells were treated with PCA (362 μM) and cultured up for different durations (12 h–96 h), then cell numbers were determined by the MTT colorimetric assay. Values are presented as mean ± SD (*n* = 8 each group).

PCA inhibited the growth of HT-29 cells in a time- and concentration-dependent manner at 48 h (Figure 2A). Treatment with 362 μM PCA for 48 h led to nearly 50% cell death (Figure 2A), and the inhibition ratio reached nearly 80% at 72 h (Figure 2B). Since interpretation of results obtained from incubations past 72 or 96 h may be difficult, treatment with 362 μM PCA for 48 h was considered optimum and used for further studies.

### 2.2. PCA Induced S-Phase Cell Cycle Arrest

**Figure 3:**
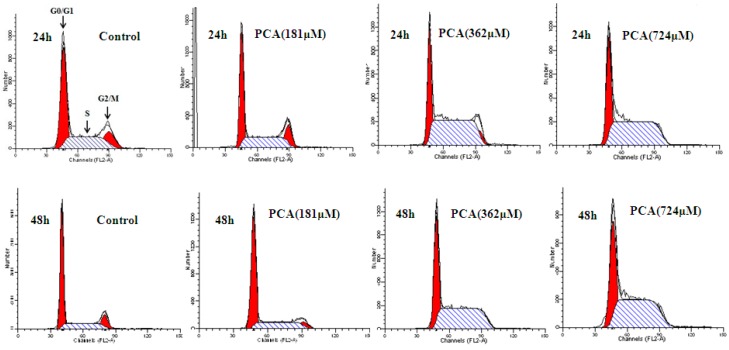

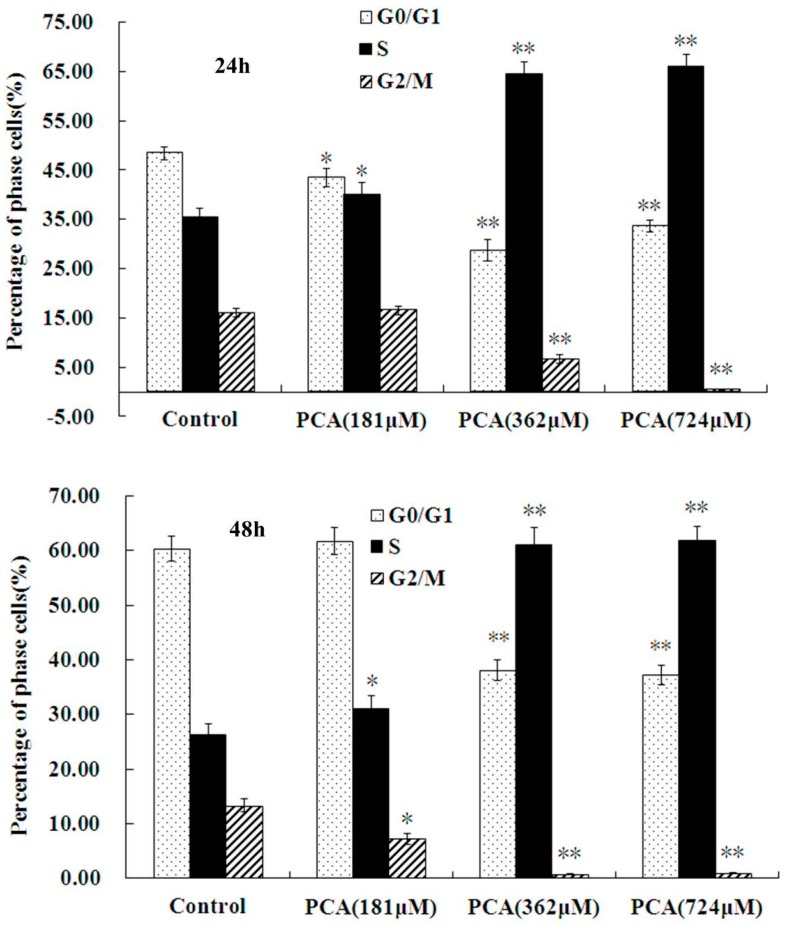
Cell-cycle (G0/G1, S and G2/M) analysis of HT-29 cells exposed without (Control) or with PCA (181, 362, 724 μM) for 24 h and 48 h. The cell-cycle distribution was based on 2N and 4N DNA content analyzed using the Cell fit software. All experiments were performed in triplicates. * *p* < 0.05, ** *p* < 0.01 vs. Control.

Flow cytometry results showed that PCA (362 μM) treatment for 48 h resulted in accumulation of cells in the S phase. The percentage of S-phase cells was 61.11% ± 3.21% in the PCA-treated group compared with that in the control group (26.23% ± 2.14%). By contrast, the proportions in G0/G1 and G2/M phases evidently decreased from 60.40% ± 2.23% and 13.36% ± 1.23% to 38.16% ± 1.89% and 0.73% ± 0.16%, respectively. These results suggested that PCA induced S-phase arrest in HT-29 cells (Figure 3).

**Figure 4:**
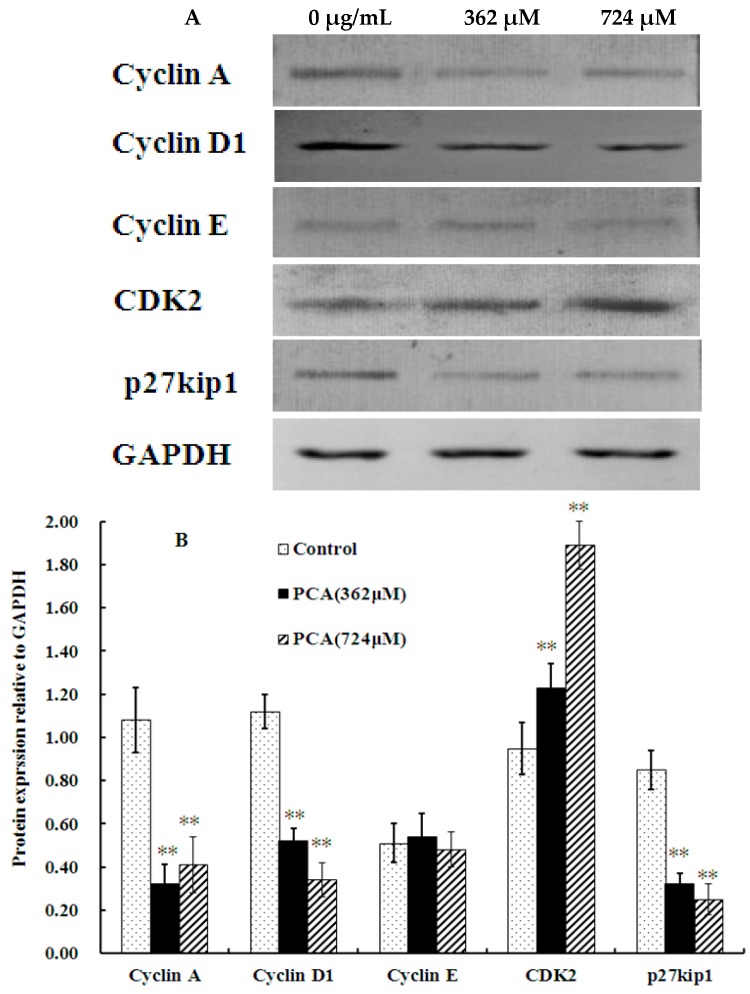
Analysis of cell-cycle related protein expression in HT-29 cells treated without (Control) and with PCA (362, 724 μM) for 48 h. (**A**) Cell lysates were subjected to SDS–PAGE; (**B**) Western blot results were quantified using Image J software (National Institutes of Health, Bethesda, MD, USA). Density values were normalized to levels of GAPDH. Data are presented as mean ± SD for three experiments per group. ** *p* < 0.01 vs. Control.

To examine the mechanism responsible for cell cycle arrest during S phase, we evaluated the expression of molecular markers associated with G1/S phase by western blot analysis. Figure 4 shows that PCA down-regulated the expression of cyclin A and cyclin D1 in HT-29 cells. PCA also remarkably increased the CDK2 protein level compared with that in the control but did not affect cyclin E expression. We also assessed the expression of p27^K^^IP1^ in cells treated with PCA. p27^KIP1^ is a cyclin-dependent kinase inhibitor that regulates cell progression from G1 to S phase [17]. Interestingly, expression level of p27^KIP1^ was significantly decreased after PCA treatment for 48 h (Figure 4). Thus, PCA inhibited HT-29 cell proliferation by down-regulating the expression of p27^KIP1^, Cyclin A, and Cyclin D1 and induced S phase arrest by up-regulating CDK2 expression.

### 2.3. PCA-Induced Apoptosis in HT-29 Cells

**Figure 5:**
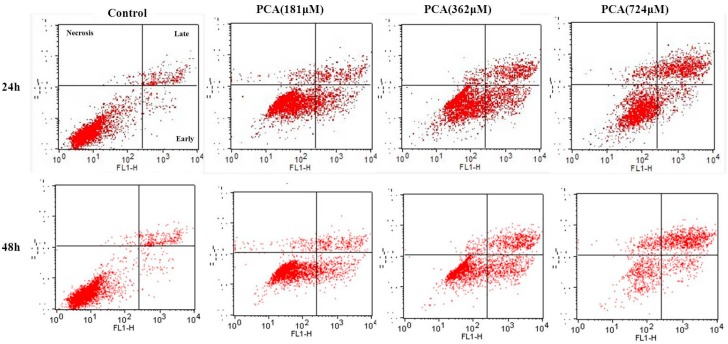

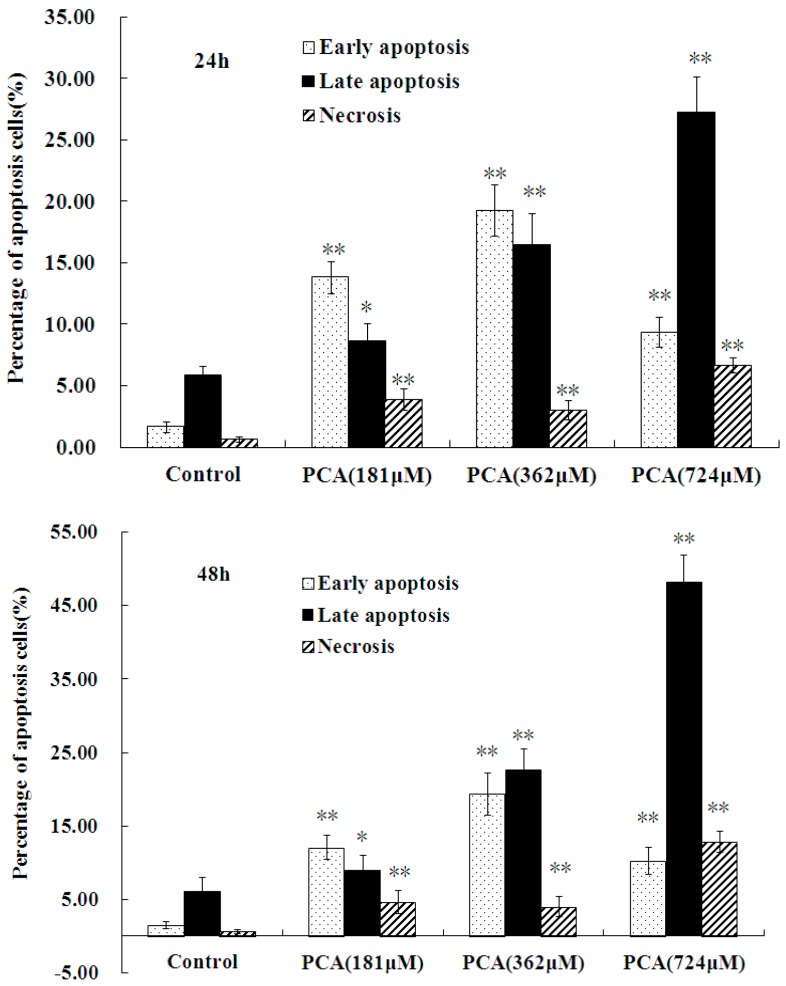
Apoptosis induced by PCA on HT-29 cells. HT-29 cells were treated without (Control) and with PCA (181, 362, 724 μM) for 24 h and 48 h. Then, they were stained with FITC-conjugated Annexin V and PI for flow cytometry. All experiments were performed in trplicates. * *p* < 0.05, ** *p* < 0.01 vs. Control.

Considering that many cytotoxic agents can induce cell cycle arrest at different phases, then result in apoptosis [16], we determined the occurrence of PCA-induced apoptosis by Annexin V-PI staining methods. Figure 5 shows that the proportion of early apoptotic cells (lower right) and late apoptotic cells (upper right) significantly increased in cells treated with PCA (0.76 μM for 48 h) from 1.51% ± 0.53% and 6.09% ± 1.87% to 10.26% ± 1.86% and 48.24% ± 3.56% in untreated cells, respectively. Moreover, the proportion of necrotic cells (upper left) significantly increased in cells treated with PCA (724 μM for 48 h) from 0.64% ± 0.39% to 12.8% ± 1.42% in untreated cells.

**Figure 6:**
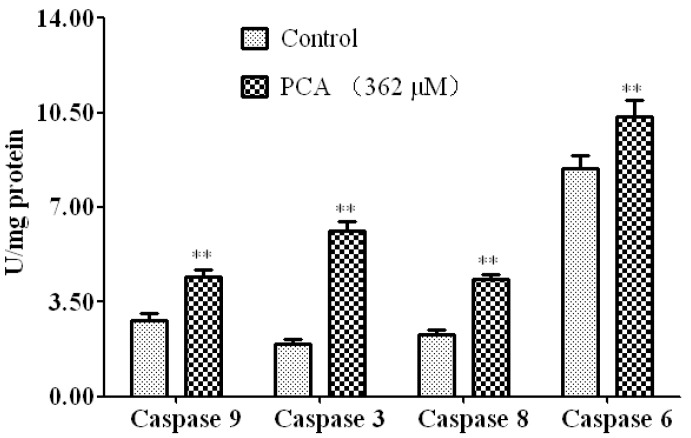
Caspases activity in HT-29 cells with or without PCA (362 μM) treatment. Data are mean ± SD three in each group ** *p* < 0.01 vs. Control.

The caspase family of proteins is believed to play a pivotal role in various apoptotic responses. Activation of the caspase cascade is an integral event in the apoptotic pathway. Therefore, the activities of caspase-9, -3, -6, and -8 in HT-29 cells treated with PCA were determined (Figure 6). Our results showed that the activity of each caspase was significantly increased in PCA-treated cells compared with that in the control group.

**Figure 7:**
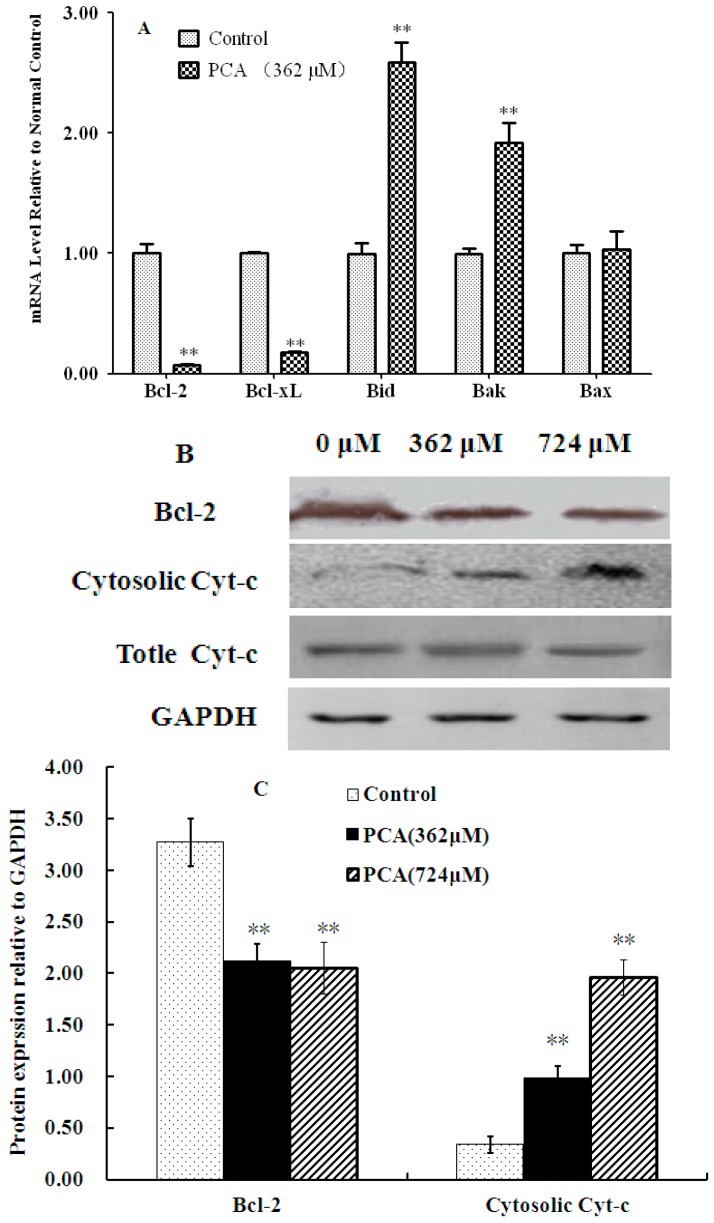
Analysis of apoptosis-related proteins in HT-29 cells. Panel (**A**) shows RT-PCR analysis of mRNA expression, Panel (**B**,**C**) show protein expression analysis in HT-29 cells. HT-29 cells were treated with different concentrations of PCA for 48 h. The density values were normalized to GAPDH. All experiments were conducted in triplicates. ** *p* < 0.01 vs. Control.

The B-cell lymphoma 2 (Bcl-2) families of proteins plays critical roles in regulating cell death via apoptosis [18,19]. To investigate the involvement of the apoptotic pathway in PCA-induced apoptosis, we examined the expression of proteins from the Bcl-2 family of genes. RT-PCR results showed that the expression of anti-apoptotic genes such as Bcl-2 and Bcl-xL significantly decreased, whereas that of proapoptotic genes, including Bid and Bak, increased in PCA-treated HT-29 cells compared with those in the normal control (Figure 7A). Western blot analysis also revealed that Bcl-2 was markedly down-regulated after PCA treatment for 48 h. Furthermore, expression level of cytosolic cytochrome c significantly increased, whereas that of total cytochrome c was not affected in PCA-treated HT-29 cells (Figure 7B,C). This indicated release of cytochrome c from HT-29 cells.

## 3. Discussion

Many chemopreventive and therapeutic agents can induce apoptosis after cell cycle arrest. PCA is a naturally occurring polyphenol found in barley [20], green Cavendish bananas [21], and grapevine leaves [22]. PCA is also one of the main compounds in *Phellinus gilvus* [8]. Exposure of human colorectal cancer cells (HCT116 and SW480 cells) to PCA suppresses cell growth and induces apoptosis in a dose-dependent manner [11]. In the present study, exposure of human colorectal cancer cells HT-29 to PCA extracted from *Phellinus gilvus* induced S-phase arrest-associated apoptosis. This phenomenon could be partly due to regulation of the p27^KIP1^-mediated cyclin-A/D1-Cdk2 signaling pathway and mitochondrial apoptotic pathway. These findings suggest that PCA has potential as an antitumor agent.

Cell growth, which reflects cell cycle progression, is aberrantly regulated in the majority of cancers [23]. Cell cycle control is therefore an important goal in treatment of cancers characterized by loss of cell cycle regulation. In the present research, PCA inhibited the growth of HT-29 cells in a time- and dose-dependent manner. To determine whether PCA treatment causes cell cycle redistribution, we analyzed the cell cycle distribution of HT-29 cells through flow cytometry. Exposure of HT-29 cells to PCA for 48 h significantly increased the number of S-phase cells (Figure 3), suggesting that PCA induced cell cycle arrest in the S phase.

Cell cycle is strictly regulated by a complex network of events, which facilitate or halt the progression of cells from one phase to the other. Cyclin-dependent kinase is a pivotal enzyme that regulates cell cycle progression. Sequential activation of kinases associated with cyclin D1, E, and A has been implicated in the control of transition from G1 to S phase [24]. When mitogenic signals stimulate a cell to enter the cell cycle, expression of D-type cyclins is increased and maintained throughout G1 as long as the growth factors are present. Cyclin E is synthesized later than D-type cyclins and peaks late in G1. The cyclin E/Cdk2 complex plays a role in G1-S transition and the initiation of DNA synthesis. The complex of cyclin A and Cdk2 is thought to function in both the initiation of DNA synthesis and the progression through S phase [25]. Numerous studies have shown that many natural agents arrest cell cycle at different phases by inhibiting cyclin A/CDK activity via down-regulation of cyclin A, D, and E subunit expression in various cancer cell lines [3,17,26]. The present results demonstrated that PCA could be a potential inhibitor of HT-29 cell proliferation and can block the entry to G2/M-phase by significantly down-regulating the level of cyclin A and D1 subunit but does not decrease cyclin E expression.

The activity of Cyclin-CDK complexes is affected by two groups of CDK inhibitors (CKIs) that exert inhibitory effects on cell cycle progression [27]. One group includes p27^KIP1^ and p21^WAF1^, which bind to all G1 cyclin/Cdk complexes, such as Cyclin A/Cdk2 and Cyclin E/Cdk2 [27,28]. p27^KIP1^ inhibits cyclin E/Cdk2 more effectively than cyclin A/Cdk2 and thus plays an important role in regulation of passage through late G1 phase [25], but the cyclin A/Cdk2 complex plays a pivotal role as an S-phase regulator, particularly during priming of DNA synthesis and its progression [29]. Evidence shows that Cdk2 activation and/or increased cyclin A expression contributes to apoptosis [30,31]. Cdk2 is activated during apoptosis, and inhibiting kinase activity reduces apoptosis [31,32]. In the present study, the protein level of CDK2 was found to be significantly upregulated in PCA-treated cells, which may be related to the down-regulation of p27^KIP1^ (Figure 4). This led us to determine the apoptosis rate of PCA-treated cells, which we found to be significantly enhanced (Figure 5).

Most anticancer agents either directly induce DNA damage or indirectly induce secondary stress-responsive signaling pathways to trigger apoptosis by activating the intrinsic (mitochondrial) apoptotic pathway. Moreover, some of these agents can simultaneously activate the extrinsic receptor pathway. Among the various proteins involved in the apoptotic machinery, the Bcl-2 family proteins are the principal regulators of the mitochondrial pathway leading to apoptosis [33]. This family of structurally-related proteins consists of pro- and anti-apoptotic members. Their pro- and anti-apoptotic activities are regulated by a range of intermolecular interactions, which converge on the outer mitochondrial membrane and result in life-or-death decisions for the cell. Pro-apoptotic Bcl-2 family members, known as “activator” proteins (Bid and Bim), can activate the “effector” proteins, namely, Bak and Bax. This causes Bak/Bax oligomerization in the mitochondria, permeabilization of the outer mitochondrial membrane, and release of factors, such as cytochrome c, which initiates the apoptotic cascade. In the present study, the expression of Bid and Bak was up-regulated in PCA-treated HT-29 cells (Figure 7A). Moreover, the protein level of cytosolic Cyt-c was found to be markedly increased. However, the total cytochrome c content was not affected in HT-29 cells treated with PCA (Figure 7B,C). This indicated that PCA induced cytochrome c release from mitochonrdia into the cytoplasm.

The pro-apoptotic function of activator proteins can be suppressed by direct binding to anti-apoptotic family members (Bcl-2 and Bcl-xL), resulting in activator sequestration and cell survival [33]. In the present study, reduced expression of Bcl-2 and Bcl-xL in PCA treated HT-29 cells may have resulted in the activation of activator proteins (Bid, Bak) and induction of HT-29 cell apoptosis.

Cytosolic Cyt-c promotes the sequential activation of caspases [34]. Apoptotic caspases can be divided into two parts: initiator (caspase-8 and caspase-9) and executioner (caspase-3 and caspase-6) caspases. Initiator caspases are apical caspases in apoptosis signaling cascades, and their activation is normally required for executioner caspase activation [35]. In the present study, activation of caspases-8, -9, -3, and -6 were significantly increased in HT-29 cells treated with PCA for 24 h. Activation of caspases-8 and -9 generally occurs before activation of caspases-3 and -7 [36]. They cleave pro-caspases-3 and -7 to activate them, and the activated caspase-3 could activate caspase-6 and commit cells to apoptotic death [36]. Caspase-9 is an indicator of the mitochondrial pathway, while Caspase-8 is a marker of the extrinsic pathway for apoptosis. In the present study, activation of both caspases-8 and -9 indicated that PCA-induced apoptosis was initiated via the death receptor as well as the mitochondrial apoptotic pathway.

In conclusion, this study revealed that PCA-mediated inhibition of HT-29 cell proliferation may be caused via S phase arrest and apoptosis. S phase cell cycle arrest and apoptosis induced by PCA were associated with p27^KIP1^-mediated cyclin-A/D1-Cdk2 signaling pathway, death receptors, and mitochondrial apoptotic pathway. The inhibitory effect of PCA on HT-29 cells as well as their associated mechanisms indicates that PCA extracted from *Phellinus gilvus* shows potential for use in colorectal cancer treatment.

## 4. Materials and Methods

### 4.1. Chemicals and Reagents

Human colorectal cancer cells, HT-29, were purchased from the Institute of Biochemistry and Cell Biology, Chinese Academy of Sciences (Shanghai, China), and grown in RPMI 1640 medium supplemented with 10% fetal bovine serum, penicillin (100 µg/mL), and streptomycin (100 μg/mL). The cells were incubated at 37 °C under a humidified atmosphere of 5% CO_2_. RPMI 1640 and 3-(4,5-dimethylthiazol-2-yl)-2,5-diphenytetrazolium bromide (MTT) were purchased from Sigma-Aldrich (St. Louis, MO, USA). First-strand cDNA synthesis kits were obtained from Fermentas (Ontario, MA, Canada). Apoptosis assay kit was purchased from Invitrogen (Carlsbad, CA, USA).

### 4.2. PCA from Phellinus gilvus

PCA was extracted from fresh-fruiting bodies of *Phellinus gilvus*. PCA was purified and identified by LC-MS (Waters, Milford, MA, USA) using the method described by Li et al. [8]. HPLC analysis indicated that the purity of PCA was more than 95%.

### 4.3. Cell Proliferation Assay 

The inhibitory effect of PCA on HT-29 cells was determined by counting the number of viable cells through an MTT-based colorimetric assay [37]. HT-29 cells (1 × 10^5^ cells/well) were treated with various concentrations of PCA (45–1448 μM) for different durations (24, 48, 72, and 96 h). Then, the cells were incubated with 50 μL of MTT solution (1 mg/mL) for another 2 h. The resulting crystals were dissolved in DMSO. Formation of formazan was measured by reading absorbance at 570 nm.

### 4.4. Cell Cycle and Apoptosis Assay

Cell cycle and apoptosis were measured using a flow cytometer by previously described methods [3,16]. HT-29 cells (3 × 10^5^ cells/well) were treated with PCA (181, 362, or 724 μM) for 24 or 48 h, then collected and washed with ice-cold PBS, then fixed in 70% ethanol. Cell cycle analyses were performed by staining with PI and the proportion of cells in different phases was calculated using the Elite Multicycle software (Phoenix Flow Systems, San Diego, CA, USA). Apoptosis was detected by staining with annexin V-FITC and PI: Annexin V−/PI+ (lower right), Annexin V+/PI+ (upper right), and Annexin V+/PI− (upper left) cells were defined as early apoptotic, late apoprotic and necrotic cells, respectively.

### 4.5. Quantitative Real-Time Reverse Transcription-PCR Analysis

HT-29 cells (1 × 10^7^) were treated with PCA (362 μM) and 0.1% DMSO for 48 h, and then harvested for RT-PCR. Total RNA was extracted using TRIZOL reagent following the supplier’s instruction. Reverse transcription was performed using a Revert Aid™ First-strand cDNA Synthesis Kit for RT-PCR by using a previously described method [38]. The primer sequences are shown in Table 1.

### 4.6. Protein Expression and Western Blot Analyses

HT-29 cells (1 × 10^7^) were treated with PCA (362 and 724 μM) for 48 h, and then collected for western blot analysis by a previously described method [38]. Total protein was measured by the bicinchoninic acid assay method. Primary antibodies (CDK2-ab6538, Cyclin A-ab7956, Cyclin E-ab7959, Cyclin D1-ab16663, p27^KIP1^-ab7961, Blc-2-ab32124, and cytochrome C-ab133504) were purchased from Abcam (Cambridge, UK).

### 4.7. Caspase Activity Assay

After treatment with 0.1% DMSO and PCA (362 μM) for 24 h, HT-29 cells (1 × 10^7^) were collected for caspase activity assay. Casapse-9, -8, -3, and -6 activities were measured using the appropriate assay kits (Beyotime Biotechnology, Shanghai, China).

### 4.8. Statistical Analysis

Data are presented as mean ± S.D. Statistical differences between control and treated groups were evaluated by Duncan’s multiple range tests. *p* < 0.05 was considered statistically significant.

## Figures and Tables

**Table 1:** Primers used in quantitative real-time reverse transcription-PCR.

Primer	Sequence 5’-3’	PCR Product Size (bp)
Bcl-2-F	GCCTTCTTTGAGTTCGGTG	91
Bcl-2-R	AGTCATCCACAGGGCGAT	
Bcl-xL-F	CCAGAAGGGACTGAATCGGA	117
Bcl-xL-R	GCATCCAAACTGCTGCTGT	
Bid-F	ATCCGTGCTGTCTCCTTTG	91
Bid-R	AAACTTCCGATGGGACCAA	
Bak-F	AACCGACGCTATGACTCAGA	111
Bak-R	GCCACTCTCAAACAGGCTG	
Bax-F	ATGGGCTGGACATTGGACTT	124
Bax-R	GCCACAAAGATGGTCACGGT	
GAPDH-F	GGTGGTCTCCTCTGACTTCAACA	135
GAPDH-R	GTTGCTGTAGCCAAATTCGTTGT	

F: Forward primer; R: Reverse primer; bp: base pairs (length of nucleic acid sequence).
